# Adipose-Derived Mesenchymal Stem Cells Promote M2 Macrophage Phenotype through Exosomes

**DOI:** 10.1155/2019/7921760

**Published:** 2019-11-05

**Authors:** June Seok Heo, Youjeong Choi, Hyun Ok Kim

**Affiliations:** ^1^Cell Therapy Center, Severance Hospital, Seoul 03722, Republic of Korea; ^2^Department of Laboratory Medicine, Yonsei University College of Medicine, Seoul 03722, Republic of Korea

## Abstract

Accumulating evidence has shown that the paracrine factors derived from mesenchymal stem cells (MSCs) are capable of regulating the immune system via interaction with various immune cells. In this study, adipose-derived MSCs (AdMSCs) and human peripheral blood monocytes (PBMCs) were isolated and cultured to examine the effects of MSC-induced macrophages (iM*Φ*) on inflammation and immune modulation. Indirect coculture with MSCs increased the expression of arginase-1 and mannose receptor (CD206), markers of activated M2 macrophages, in the PBMCs demonstrating that MSC-secreted factors promoted M2-M*Φ* polarization. Additionally, iM*Φ* exhibited a similar higher inhibitory effect on the growth of activated T cells compared to that in the other groups (AdMSCs only, AdMSCs plus iM*Φ*), implying that iM*Φ* can play a sufficient functional role. Interestingly, the population of FoxP3 Treg cells significantly increased when cocultured with iM*Φ*, suggesting that iM*Φ* have an immunomodulatory effect on the Treg cells through the modulation of the FoxP3 expression. Notably, iM*Φ* expressed high levels of immunosuppressive and anti-inflammatory cytokines, namely IL-10 and TSG-6. Furthermore, we confirmed that the AdMSC-derived exosomes modulated macrophage polarization by upregulating the expression of M2 macrophage markers. Conclusively, our results suggest that iM*Φ* play a significant role in regulating the immunomodulatory- and inflammatory-mediated responses. Thus, iM*Φ* may be used as a novel stem cell-based cell-free therapy for the treatment of immune-mediated inflammatory disorders.

## 1. Introduction

Mesenchymal stem cells (MSCs) regulate immunomodulatory and anti-inflammatory effects in diverse ways in response to the specific niche or microenvironments [1]. Numerous studies have shown that the MSCs modulate immune responses through a variety of mechanisms by interacting with the immune cells [2, 3]. MSCs, therefore, have a great therapeutic potential for the treatment of inflammatory diseases. Until now, the clinical applications of MSCs derived from various tissues, such as adipose tissue and bone marrow, were being aggressively examined for the treatment of diverse disorders including intractable diseases [4]. Further, bioactive molecules secreted by MSCs have been considered the main treatment strategy rather than cell engraftment and differentiation since they exhibit diverse therapeutic effects in diseases such as arthritis and liver injury [5].

Macrophages possessing high plasticity promote tissue regeneration, mediate immunomodulation, and regulate cell proliferation in response to specific environments [6]. Macrophages that play critical roles in immunity are usually divided into two subtypes, the immune-reactive or proinflammatory M1 (classically activated macrophages) and immune-suppressive or anti-inflammatory M2 (alternatively activated macrophages) [7]. The alternatively activated M2 macrophages play a pivotal role in regulating the immune system and tissue remodeling such as during wound healing [8]. MSCs are known to stimulate macrophages to produce anti-inflammatory and immunosuppressive cytokines such as interleukin- (IL-) 10, and thereby induce polarization toward an M2 subtype expressing CD206 [9]. Li et al. revealed that the human umbilical cord-derived MSCs induce M2 polarization of macrophages *in vivo* [10]. Several studies have focused on the effects of MSCs on the immune cells including macrophages, T lymphocytes, dendritic cells, and natural killer cells; however, very little is known regarding the cross-talk between adipose-derived MSCs (AdMSCs) and macrophages [11]. Therefore, it is critical to have a better understanding of the effects of AdMSCs on macrophages for developing effective treatment strategies in the future. Here, we hypothesized that the interaction between macrophages and AdMSCs induces M2 polarization.

Among the various factors responsible for the therapeutic effects of MSCs, exosomes have been recently described as key mediators for transferring proteins, DNAs, RNAs, and lipids to other cells for communication [12]. Thus, we surmised that AdMSC-derived exosomes are powerful players to influence processes involved in macrophage M2 polarization. Increasing numbers of studies have shown that MSCs affect the activation, plasticity, and functionality of macrophages in a cell contact-dependent or contact-independent manner [10]. In the present study, peripheral blood mononuclear cells (PBMCs) and AdMSCs were indirectly cocultured using the transwell system in order to investigate the effects of exosomes released by AdMSCs on macrophages. In other words, we evaluated whether M2 polarization could be induced by the secreted exosomes. Herein, we found that the AdMSC-derived exosomes acted as mediators and promoted the propagation of M2 macrophages *in vitro*. These findings will contribute to the generation of anti-inflammatory M2 macrophages by exosomes for future stem cell-based cell-free therapy against inflammation-related diseases.

## 2. Materials and Methods

### 2.1. Culture of Adipose-Derived MSCs

Human AdMSCs were isolated from healthy donors with approval from the research ethics committee of Severance Hospital of Yonsei University, Seoul, Republic of Korea, following informed consent (approval no. 4-2019-0060). The isolated mononuclear cells were cultured in DMEM- (Dulbecco's Modified Eagle Medium-) low glucose (Invitrogen, Carlsbad, CA, USA) supplemented with 10% FBS (Invitrogen), 100 U/ml penicillin (Invitrogen), and 100 *μ*g/ml streptomycin (P/S, Invitrogen) of 1 × 10^6^ cells in a 75 cm^2^ tissue culture flask (Nunc, Roskilde, Denmark) at 37°C with 5% humidified carbon dioxide. The culture medium was exchanged with a fresh medium after 24 h to remove nonadherent cells and cell debris. The medium was changed every 3 or 4 days until the attached cells exhibited the spindle-shaped morphology of MSCs. Upon reaching approximately 90% confluence, the cells were harvested using 0.05% trypsin/EDTA (Invitrogen) and were subcultured at a 1 : 3 or 1 : 4 ratio for cell proliferation.

### 2.2. Differentiation Assay

To assess whether AdMSCs have the capacity of differentiating into osteoblasts, adipocytes, and chondrocytes, the cultured AdMSCs were induced with a specific differentiation media kit (Lonza, Allendale, NJ, USA). Briefly, the culture media were replaced with an osteogenic or chondrogenic medium when the cells reached 70% confluence. To differentiate the AdMSCs into chondrocytes, 10 ng/ml of transforming growth factor-*β*3 was added to the cells when media were changed. For adipogenic differentiation, the cells were cultured with adipogenic differentiation media when they reached 100% confluence. The media were changed every 3-4 days. After 2 weeks, osteogenesis was evaluated by von Kossa staining. Chondrogenic differentiation was analyzed by safranin-O staining. Adipogenesis was determined by staining the cells with Oil red O. Images of the stained cells were taken using an Olympus IX71 microscope (Olympus, Tokyo, Japan).

### 2.3. FACS Analysis

To analyze surface markers, the cultured AdMSCs were isolated and harvested at a single-cell level. Then, the cells were stained with fluorescein isothiocyanate- (FITC-) conjugated or phycoerythrin- (PE-) conjugated monoclonal antibodies (BD Biosciences Pharmingen, San Diego, CA, USA). FITC- and PE-conjugated isotype antibodies were used as negative controls. The fluorescence stain-conjugated monoclonal antibodies used in the study were anti-CD34, anti-CD45, anti-CD73, anti-CD90, and anti-CD105 (all from BD Biosciences). Data were obtained using the Cytomics Flow Cytometer (Beckman Coulter, Fullerton, CA, USA). For CD206 analysis, cells were stained with CD206 antibody (Abcam, Cambridge, MA, USA), and rabbit isotype IgG (Alexa Fluor 594, Invitrogen) was used as the negative control. Acquisition was performed with BD FACS Aria III (BD Biosciences Pharmingen).

### 2.4. Coculture of AdMSCs with PBMCs

For coculture, AdMSCs were seeded at 1 × 10^5^ cells in a 12-well plate (BD Falcon, USA). PBMCs were obtained from three healthy donors. Briefly, mononuclear cells from the blood were separated by centrifugation using a Ficoll-Hypaque gradient (GE Healthcare, Uppsala, Sweden) and suspended in the RPMI medium containing 10% FBS and 1% P/S (all from Invitrogen). PBMCs were plated onto transwell cell culture inserts (Corning, NY, USA) at an AdMSC to PBMC ratio of 1 : 20 and cocultured for 24 h. The cells were maintained with RPMI supplemented with 10% FBS and 1% P/S during the 24 h culture period. The culture groups were as follows: PBMCs alone, AdMSCs alone, induced macrophages (iM*Φ*) alone, PBMCs+AdMSCs, and AdMSCs+iM*Φ*.

### 2.5. Quantitative PCR

Total RNA was extracted using RiboEx reagent (GeneAll, Seoul, Korea). The RNA was reverse transcribed into cDNA using Maxime RT PreMix (iNtRON, Seongnam, Korea), according to the manufacturer's instructions. Quantitative PCR was performed in 96-well plates using the LightCycler 480 SYBR Green I Master mix (Roche Molecular Systems, Pleasanton, CA, USA) on a LightCycler 480 System (Roche) under the following conditions: 95°C for 5 min and 95°C for 10 s, 45 cycles of 60°C for 20 s, and 72°C for 15 s. The sequences of the primers used are listed in Table 1. Gene expression was normalized to that of GAPDH and analyzed using advanced relative quantification based on the E-method provided by Roche Applied Science. The data are expressed as the mean ± SD of three independent experiments.

### 2.6. Immunostaining

Cells were fixed in 4% paraformaldehyde (Biosesang, Seongnam, Korea) after washing with PBS (Invitrogen) containing 0.1% bovine serum albumin (BSA, Sigma Chemical Co., St. Louis, MO, USA). After 20 min on ice, the cells were washed with PBS/0.1% BSA twice for 10 min. Next, permeabilization solution containing 0.3% Triton X-100 (Sigma) was added to the cells. After 5 min, primary antibody against CD206 (Abcam) was dispensed at dilution of 1 : 200. After incubating overnight at 4°C, the fluorescence-labeled anti-rabbit IgG (Invitrogen) at dilution of 1 : 400 was used as the secondary antibody for 40 min at RT. After washing twice for 10 min, the cells were costained with DAPI (Sigma) for 5 min. The number of CD206-stained cells was counted and fluorescence images were obtained using a fluorescence microscope (Olympus IX71, Tokyo, Japan). The data are expressed as the mean ± SD of three independent experiments.

### 2.7. T Cell Proliferation Assay

To evaluate the ability of cells to suppress activated T cell proliferation, PBMCs (2 × 10^6^) were cocultured with cells of each type (1 × 10^5^) in a 12-well plate including transwell inserts. The attached cell groups were as follows: 1 × 10^5^ of AdMSCs alone, 1 × 10^5^ of iM*Φ* alone, and 5 × 10^4^ of AdMSCs plus 5 × 10^4^ of iM*Φ*. To activate the T cells, 10 *μ*g/ml of phytohemagglutinin (Sigma) was added for 72 h during the coculture. To investigate the inhibition of T cells, proliferation assay was performed on the harvested cells using a WST-based proliferation assay kit (EZ-Cytox, Daeil Lab, Seoul, Korea), according to the manufacturer's protocols. WST produces a water-soluble formazan dye upon reduction, in the presence of an electron carrier. WST, being nonradioactive, can be used in sensitive colorimetric assays for the determination of the number of viable cells during cell proliferation. Activated T cells alone were used as the control. The data are expressed as the mean ± SD of three independent experiments.

### 2.8. Isolation of Exosomes from AdMSCs

To isolate the exosomes from cultured AdMSCs, exosomes were extracted from AdMSC culture media using an exosome isolation kit (System Biosciences, California, USA), according to the manufacturer's instructions. Briefly, 5 ml culture media was transferred to a 15 ml conical tube. After centrifugation at 1500 × *g* for 5 min at RT, the media supernatant was transferred to a 15 ml conical tube. Thereafter, 1 ml of ExoQuick-TC reagent was added to the supernatant and mixed by inverting the tube four times. After incubation at 5°C overnight, the mixture was centrifuged at 1500 × *g* for 30 min at RT. After removing the supernatant, the exosomes were resuspended in PBS. Finally, the exosomes were stored at -80°C after quantification using the BCA protein assay kit (Invitrogen). Then, 5 *μ*g/ml of exosomes was added to the experimental test groups. To identify exosomes, the morphology of the isolated exosomes was analyzed by transmission electron microscopy (JEM-1011, JEOL, Japan). Briefly, a formvar-carbon-coated EM grid was placed formvar-side down on top of an exosome drop for approximately 1 min. The grid was removed, blotted with filter paper, and placed onto a drop of 2% uranyl acetate for 15 s. The excess uranyl acetate was removed. One representative of three independent experiments is shown.

### 2.9. Western Blot Assay

Total protein was extracted using RIPA buffer (Biosesang). The proteins (50 *μ*g) were separated on 12% gradient-precast gels by sodium dodecyl sulfate polyacrylamide gel electrophoresis and transferred onto PVDF membranes (Bio-Rad Laboratories, Redmond, WA, USA). After blocking with 5% BSA in TBST, the membranes were incubated with the primary antibodies CD9 (Abcam, 1 : 500) and CD63 (Abcam, 1 : 500) at 4°C overnight. Thereafter, the membranes were incubated with HRP-conjugated anti-mouse and anti-rabbit IgG (1 : 1000, GeneTex, CA, USA), respectively, at room temperature for 1 h. The secondary antibodies were detected using the LAS 4000 mini system (GE Healthcare, Uppsala, Sweden).

### 2.10. Statistical Analysis

All data are expressed as the mean ± SD. Statistical comparisons were performed by one-way ANOVA with post hoc Bonferroni's correction and by Student's *t* test. A *P* value < 0.05 was considered to indicate a statistically significant difference. All statistical analyses were performed using SPSS software 18 (SPSS Inc., Chicago, IL, USA).

## 3. Results

### 3.1. AdMSCs Increased the Expression of M2 Macrophage Markers

**Figure 1:**
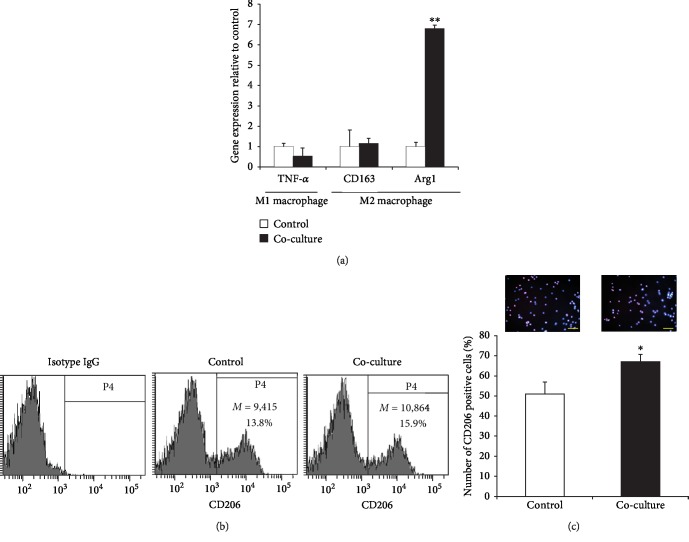
AdMSCs induce macrophage M2 polarization. (a) qPCR analysis of coculture of AdMSCs with PBMCs for 24 h revealed increased gene expression of *Arg1* (M2 macrophage marker). (b) Expression of CD206 in PBMCs after coculture as assessed by flow cytometry. (c) PBMCs were cocultured with AdMSCs for 24 h, then stained with CD206 (M2 macrophage marker) antibody by immunofluorescent staining (scale bar = 100 *μ*m). The percentage of CD206-positive cells was determined by counting the number of stained cells. The data are expressed as the mean ± SD of three independent experiments. ^∗^*P* < 0.05, ^∗∗^*P* < 0.01.

PBMCs (2 × 10^6^) were cocultured with AdMSCs (1 × 10^5^) using a transwell system with the RPMI medium supplemented with 10% FBS and 1% P/S. After overnight incubation, the expression of macrophage markers was analyzed. The coculture group showed that the expression of M2 macrophage marker Arg1 increased significantly. However, the expression of TNF-*α* (M1 marker) and CD163 (M2 marker) did not change significantly (Figure 1(a)).

To confirm whether AdMSCs induce M2 phenotype in PBMCs, we evaluated the expression rate of CD206 (mannose receptor), a specific marker of M2 macrophages, using flow cytometry and immunofluorescence staining. The coculture slightly promoted the CD206 expression of M2 macrophages (control: 13.8%; coculture: 15.9%) (Figure 1(b)). Furthermore, the results of immunostaining showed that coculture with AdMSCs increased the percentage of CD206-positive cells (Figure 1(c)). Thus, these results demonstrated that AdMSCs induce M2 phenotype in PBMCs.

### 3.2. AdMSC-Induced Macrophages Have Immunosuppressive and Anti-inflammatory Effects

**Figure 2:**
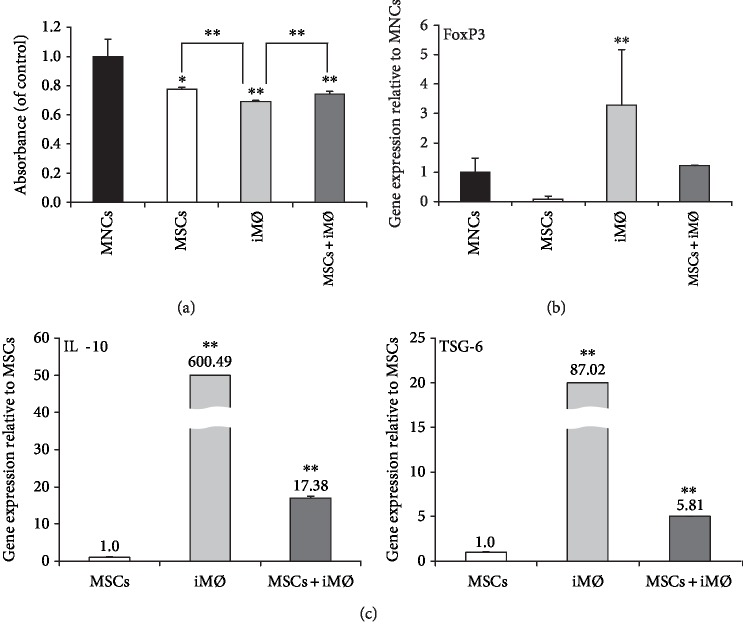
Immunomodulatory effects on activated T cells cocultured with AdMSCs, iM*Φ*, and AdMSCs+iM*Φ*. (a) Proliferation of PBMCs cocultured with various cells for 72 h was analyzed by a WST-based proliferation assay kit to examine suppression of PBMCs by the different cells. (b) Relative gene expression of FoxP3 was significantly increased in iM*Φ*-alone culture condition. (c) The relative gene expression levels of *IL-10* and *TSG-6*, immunomodulatory- and anti-inflammatory-related factors, were evaluated by qPCR. The data are expressed as the mean ± SD of three independent experiments. ^∗^*P* < 0.05, ^∗∗^*P* < 0.01.

The purpose of this experiment was to examine whether iM*Φ* induced by AdMSCs are effective as AdMSCs. To assess the immunosuppressive effects, activated T cells were cocultured with AdMSCs, iM*Φ* (AdMSC-induced M*Φ*), and AdMSCs plus iM*Φ*. PBMCs were used as the control to evaluate immunosuppressive activity. The proliferation activity of T cells was determined using the WST-based EZ-Cytox assay after 72 h. T cell proliferation was inhibited by all the cell groups. Importantly, iM*Φ* strongly suppressed cell proliferation (Figure 2(a)).

Immunosuppression is mediated by various immune cells, including regulatory T cells. The expression of FoxP3, an immune-modulating gene, greatly increased in iM*Φ*, supporting the observation of the inhibition of T cell proliferation (Figure 2(b)). We next analyzed the gene expression of *IL-10* and *TSG-6*, molecules associated with anti-inflammation, by qPCR. Notably, the expression of *IL-10* and *TSG-6* was higher in the iM*Φ* than in the AdMSCs and AdMSCs plus iM*Φ*, indicating that iM*Φ* alone could exert powerful immunosuppressive and anti-inflammatory activity (Figure 2(c)).

### 3.3. Characterization of AdMSC-Derived Exosomes

**Figure 3:**
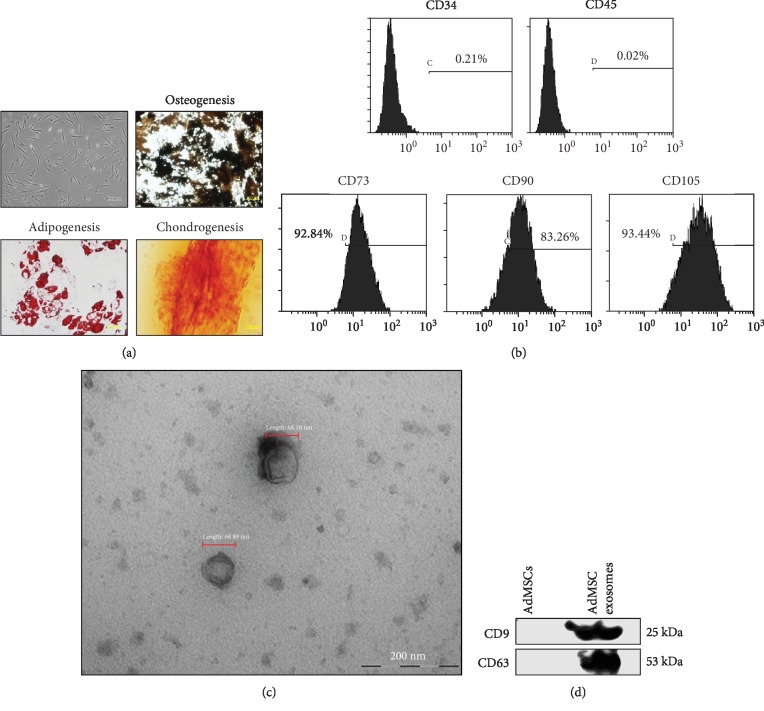
Isolation and characterization of AdMSCs and AdMSC-derived exosomes. (a) Morphology of cultured AdMSCs displayed the typical spindle shape of MSCs (magnification, 100x). Under specific differentiation conditions, AdMSCs were differentiated into osteoblasts (magnification, 200x), adipocytes (magnification, 400x), and chondrocytes (magnification, 200x). (b) Flow cytometry histograms show the positive or negative immunophenotype of cultured AdMSCs. The cells expressed CD73, CD90, and CD105, known as MSC markers. (c) Morphology of exosomes was identified by transmission electron micrograph (TEM). Scale bar = 200 nm. (d) Protein expression of CD9 and CD63, the exosomes markers, was analyzed by western blot assay.

After primary isolation, AdMSCs were cultured to obtain the exosomes. Cultured AdMSCs exhibited a typical spindle-shaped morphology and differentiation capacity for osteogenesis, adipogenesis, and chondrogenesis (Figure 3(a)). The AdMSCs expressed the MSC markers, namely CD73, CD90, and CD105, as assessed by flow cytometry (Figure 3(b)). Exosomes isolated from AdMSCs were analyzed by TEM. The results showed that exosomes were cup-shaped, membrane-bound vesicles with a diameter of 67 nm (Figure 3(c)). CD9 and CD63, the specific marker proteins for exosomes, were strongly detected in the isolated AdMSC exosomes as demonstrated by western blot assay, whereas these markers were not detected in AdMSCs (Figure 3(d)).

### 3.4. AdMSC-Derived Exosomes Increased M2 Macrophage Marker Expression by Activating M2 Macrophage Transcription Factors

**Figure 4:**
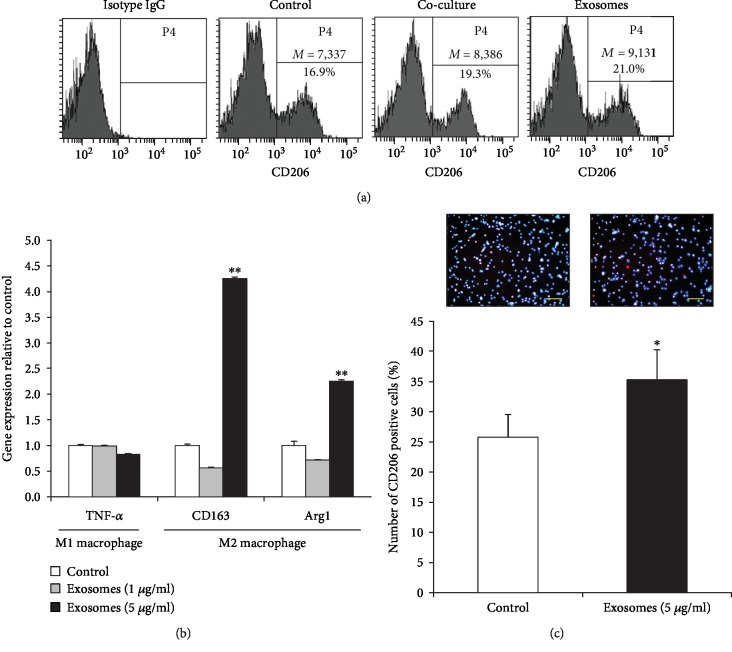
AdMSC-derived exosomes induce macrophage M2 polarization. (a) Expression of CD206 in PBMCs as assessed by flow cytometry. (b) qPCR analysis of PBMCs treated with 5 *μ*g/ml exosomes for 24 h showed increased gene expression of *CD163* and *Arg1* (M2 macrophage markers). (c) M2 macrophage polarization by exosomes was confirmed by determining the percentage of CD206-stained cells (scale bar = 100 *μ*m). The data are expressed as the mean ± SD of three independent experiments. ^∗^*P* < 0.05, ^∗∗^*P* < 0.01.

We investigated M2 polarization by flow cytometry analysis. The mean value of CD206-positive cells in cocultured and exosome-treated groups was higher than that in the control (Figure 4(a)). The results suggest that AdMSC-derived exosomes could successfully induce M2 macrophages.

In order to further assess whether AdMSC-derived exosomes induce M2 macrophages, 1 *μ*g/ml and 5 *μ*g/ml exosomes were added to the PBMCs for 24 h. As shown in Figure 4(b), the mRNA expression levels of CD163 and Arg1 (M2 macrophage markers) significantly increased in the 5 *μ*g/ml exosome-treated PBMCs. To confirm the presence of M2 phenotype, the cells treated with 5 *μ*g/ml exosomes were evaluated for CD206, an M2 macrophage-specific marker, by immunofluorescence staining. Taken together, these results showed that the percentage of CD206-positive cells was increased in 5 *μ*g/ml exosome-treated PBMCs (Figure 4(c)).

**Figure 5:**
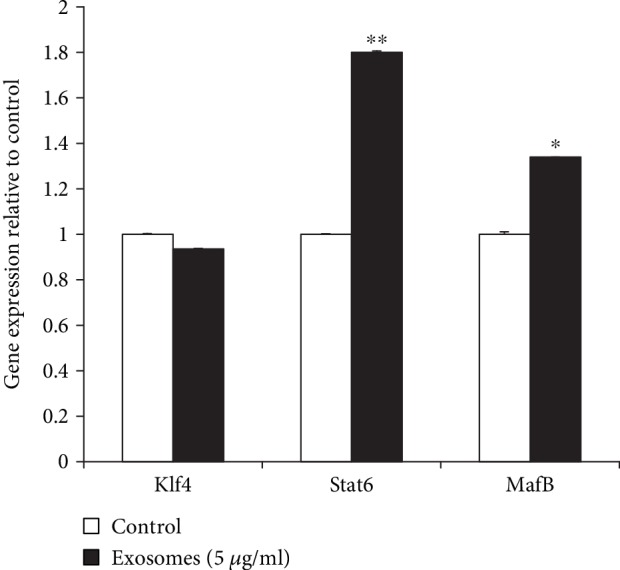
AdMSC-derived exosomes activate macrophage M2 polarization-related transcription factors. PBMCs treated with exosomes showed significant effects on the activity of transcription factors Stat6, MafB, and Klf4 of M2 macrophages. The data are expressed as the mean ± SD of three independent experiments. ^∗^*P* < 0.05, ^∗∗^*P* < 0.01.

We next examined the effects of AdMSC-derived exosomes on inducing M2 macrophage phenotype by analyzing the expression of M2 macrophage-specific transcription factors, namely Klf4, Stat6, and MafB.  Figure 5 reveals significant activation of Stat6 and MafB in the exosome treatment group, although the expression level of Klf4 did not show a significant increase. Collectively, these data suggest that AdMSC-derived exosomes sufficiently induce M2 phenotype in PBMCs.

## 4. Discussion

Transplantation using MSCs for cell therapy shows positive effects in various diseases such as myocardial infarction and multiple system atrophy; however, no transdifferentiation to tissue-specific cells was observed *in vivo* [13, 14]. This means that the beneficial effects of MSCs occur via paracrine factors, including exosomes and secreted molecules. Accumulating evidence has supported that MSCs promote tissue repair by reducing inflammation via their paracrine effects [15]. In terms of safety, tumor formation and chromosomal abnormalities have been reported after transplantation of MSCs in experimental models *in vivo* [16]. Based on the abovementioned studies, recent efforts have shown an increasing number of positive outcomes employing the paracrine activity of MSCs without cell engraftment [17].

Recently, Ulivi et al. showed that the MSCs are able to shift the polarization of proinflammatory M1 macrophages toward an anti-inflammatory M2 phenotype, by secreting cytokines and soluble factors [18]. Additionally, Naoki Takizawa et al. showed that cell-to-cell direct coculture of MSCs and blood cells promoted M2 macrophage polarization [19]. We herein examined the propagation of M2 macrophages from PBMCs, in an indirect coculture system. Quantitative PCR analysis revealed that the monocytes cocultured with AdMSCs significantly expressed Arg1, which is a known M2 macrophage marker. Moreover, immunofluorescence staining analysis at the protein level confirmed that most of the AdMSC-induced macrophages (iM*Φ*) were positive for CD206, which in turn implied that the molecules secreted by AdMSCs by indirect contact play an important role in the expansion of M2 markers in the polarized macrophages. However, we not only failed to reduce the expression of TNF-*α* on the surface of M1 macrophages but also promoted the expression of CD163 on the surface of M2 macrophages. Soluble factors secreted by MSCs promote the early expression of CD163 on M2 macrophages, which indicates that the interaction time and proportion of cells are critical factors in the expression of macrophage markers [20]. These results are consistent with the conclusion of Chen et al. that molecules produced by MSCs had no significant effect on the levels of TNF-*α* [20]. The reason behind this may be that there are intercellular interactions of unknown factors between AdMSCs and macrophages. Additionally, another possibility is that molecules secreted by AdMSCs may not induce complete macrophage polarization. Thus, cell populations affected by AdMSCs were expressed as induced macrophages (iM*Φ*) in our study.

To investigate the effects of iM*Φ* on immunomodulation and anti-inflammation, an activated T cell coculture system was employed. All the cell groups including AdMSCs, iM*Φ*, and AdMSCs plus iM*Φ* inhibited T cell proliferation. Notably, out of all of the cells, iM*Φ* strongly suppressed T cell proliferation, revealing that iM*Φ* alone has the greatest immunosuppressive potency. In other words, exosomes from AdMSCs could be applicable for use as immunosuppressive agents by inducing M2 macrophage phenotype *in vivo*. Regulatory T cells expressing *FoxP3* gene are generally immunosuppressive and inhibit the proliferation of T cells [21]. Results of qPCR showed that the expression of *FoxP3* greatly increased in PBMCs cocultured with iM*Φ*, indicating that iM*Φ* have a powerful modulatory effect on FoxP3-positive T cells. However, in terms of immunosuppression, the interactive possibility of other immune cells cannot be excluded. IL-10 and TSG-6 are the main anti-inflammatory factors expressed in M2 macrophages [22]. In the present study, iM*Φ* exhibited the highest increase in the expression levels of IL-10 and TSG-6 as compared to those in other AdMSCs and AdMSCs plus iM*Φ*. Taken together, iM*Φ* has greater immunomodulatory and anti-inflammatory potential as compared to the AdMSCs.

We next explored the molecules through which AdMSCs mediated the induction of M2 macrophages. More recently, Zhao et al. reported that the exosomes from AdMSCs attenuate inflammation and obesity via M2 macrophages in adipose tissue [23]. Exosomes are the key players that are capable of communicating with other cells and modulate characteristics through various signaling pathways by transferring intercellular information, including RNAs, proteins, and DNAs [24]. Therefore, recently, exosomes have been applied in diverse biological fields, such as stem cell biology, cancer biology, regenerative medicine, and immunology. In this study, we explored whether the AdMSC-derived exosomes might affect M2 macrophages induction. The ultimate goal of this study was to determine whether M2 macrophages were induced by AdMSC-derived exosomes. Further, we have tried to confirm the possibility of stem cell-based cell-free therapy using our results. Interestingly, AdMSC-derived exosomes significantly induced M2 macrophage phenotype in PBMCs by upregulating CD163, Arg1, and CD206, suggesting that the exosomes indeed have the potential to act as a next-generation therapeutic tool in the field of stem cells.

Wnt/*β*-catenin and notch signaling are known to promote M2 macrophage polarization and signaling through IL-4 and IL-13 [25–27]. Yang et al. reported that the testes-specific protease 50 modulates macrophages and induces M2 polarization via TNF-*α*, IL-1*β*, and NF-*κ*B signaling pathways [28]. Recently, the transcription factors MafB, Klf4, and Stat6 have been reported to promote M2 polarization by inducing the genes related to anti-inflammatory functions [29–31]. Our data show that the AdMSC-derived exosomes induce M2 polarization in macrophages by activating MafB and Stat6. However, a significant change in *Klf4* gene expression was not observed, indicating that Klf4 may not be a key transcription factor in stimulating M2 macrophages, and interaction with other factors may play definitive roles. Additional detailed studies are necessary to assess the functions of secreted exosomes. In addition, the signaling pathways and/or mechanisms activating the transcription factors for M2 macrophage polarization remain to be elucidated. Furthermore, a comparative analysis of various molecules, including exosomes and microvesicles secreted by the AdMSCs, needs to be performed by using RNA arrays and bioinformatics. We cannot exclude the effects of considerable donor variations because we observed variation within the same type of PBMCs.

## 5. Conclusions

In summary, this study reveals that AdMSCs could induce M2 macrophages through exosomes, and these effects might be through the activation of Stat6 and MafB transcription factors, although the effects were not confirmed at protein levels. Our results support two conclusions. Firstly, AdMSCs induce monocytes toward an M2 phenotype possessing immunomodulatory and anti-inflammatory functions, in a cell contact-independent manner. Secondly, the AdMSC-derived exosomes inducing M2 polarization could be safely used as a useful stem cell-based cell-free tool for the clinical treatment against inflammatory diseases. Furthermore, therapies using exosomes could overcome the regulatory hurdles and clinical risks such as tumor formation by transplantation of stem cells. Future research will determine whether exosomes secreted by AdMSCs have anti-inflammatory functions in inflammation-related disease models such as wound healing.

## Figures and Tables

**Table 1:** Primer sequences.

Gene	Primer sequence (5′-3′)
*TNF-α*	Forward: TTGAGGGTTTGCTACAACATGGG Reverse: GCTGCACTTTGGAGTGATCG

*CD163*	Forward: CGGCTGCCTCCACCTCTAAGT Reverse: ATGAAGATGCTGGCGTGACA

*Arg1*	Forward: ACAGTTTGGCAATTGGAAGCA Reverse: CACCCAGATGACTCCAAGATCAG

*FoxP3*	Forward: TCATCCGCTGGGCCATCCTG Reverse: GTGGAAACCTCACTTCTTGGTC

*IL-10*	Forward: ACCTGGTAGAAGTGATGCCCCAGGCA Reverse: CTATGCAGTTGATGAAGATGTCAA

*TSG-6*	Forward: GGTGTGTACCACAGAGAAGCA Reverse: GGGTTGTAGCAATAGGCATCC

*Stat6*	Forward: CCTTGGAGAACAGCATTCCTGG Reverse: GCACTTCTCCTCTGTGACAGAC

*MafB*	Forward: GACGCAGCTCATTCAGCAG Reverse: CCGGAGTTGGCGAGTTTCT

*Klf4*	Forward: ACCAGGCACTACCGTAAACACA Reverse: GGTCCGACCTGGAAAATGCT

*GAPDH*	Forward: ACCCACTCCTCCACCTTTGA Reverse: CTGTTGCTGTAGCCAAATTCGT

## Data Availability

The data used to support the findings of this study are available from the corresponding author upon request.
